# Using Virtual Reality for Perioperative Nursing Education in Complex Neurosurgical Surgeries: A Feasibility and Acceptance Study

**DOI:** 10.7759/cureus.55901

**Published:** 2024-03-10

**Authors:** Linda Nguyen, Martina Bordini, Clyde Matava

**Affiliations:** 1 Nursing, Hospital for Sick Children, Toronto, CAN; 2 Anesthesia and Pain Medicine, Hospital for Sick Children, Toronto, CAN; 3 Department of Medical and Surgical Sciences (DIMEC), University of Bologna, Bologna, ITA

**Keywords:** feasibility studies, nursing education and practice, simulation in medical education, virtual augmented reality, virtual reality in medical education

## Abstract

Background

Operating room (OR) nurses' training for surgical fields such as neurosurgery is often inconsistent and overly lengthy due to the lack of consistently scheduled procedures and the nature of procedures being for the most part emergencies. Virtual reality (VR) simulation has been explored for nurses training in various contexts with positive results.

Objectives

To develop a VR simulation that could replicate a pediatric neurosurgery craniotomy procedure reflecting a real OR scenario and the surgical procedural sequence of a craniotomy; and to assess OR nurses’ confidence in assisting craniotomy procedures as scrub nurses before and after the VR simulation.

Methods

A pediatric craniotomy procedure was replicated using VR technology by a collaborative partnership between education, content, and technology experts within the Hospital for Sick Children, Toronto. Self-confidence among OR nurses to assist in craniotomy procedures was explored pre- and post-VR training sessions with a questionnaire ideated by the authors evaluating knowledge relevant to assisting craniotomy procedures with seven items.

Results

In total, 7 OR nurses participated in the study. The post-VR sessions questionnaires showed an increase of positive answers “extremely comfortable with the procedure” and “moderately comfortable with the procedure” compared to pre-VR sessions in all items except for “identify the hemostatic agents required during a bleed,” for which no difference was noted. There were no issues with the equipment.

Conclusion

VR simulation session is an acceptable model to train OR nurses for the scrub nurse role in craniotomy procedures. VR simulation is a practical learning strategy for clinical situations that may occur inconsistently in real-time practice.

## Introduction

Perioperative nursing education is challenged by several factors. Among them, the expectation of nurses to be competent across multiple surgical specialties, the need for highly specialized skills, and limited opportunities for real-time training have been identified [1]. The preparedness of nurses working in specialized settings, such as the operating room (OR), has been found to be hindered by limited access to relevant training opportunities. Moreover, factors such as high patient acuity, time pressure, and the presence of multiple disciplines working within proximity create an environment that is not conducive to learning. Challenges in the learning process for nurses can lead to lowered self-esteem, poor retention, and job dissatisfaction [2].

The current training model for OR nurses is dependent on surgical cases as they arise. This strategy is unpredictable and inconsistent and results in lengthy orientations. These problems are particularly prevalent in surgical fields like neurosurgery, due to the lack of consistently scheduled procedures. In our institution, approximately 40-50% of neurosurgery cases are emergencies, which makes it difficult to organize education and maintain clinical competencies. Severely limited training in neurosurgery has resulted in a lack of confidence among OR nurses and in an unwillingness to assist in these surgeries, particularly as a scrub nurse [3].

VR is a tool that provides a human-computer interface that embeds the users in a three-dimensional simulated alternative reality. The virtual world provides an immersive multisensory experience that combines auditory, visual, and haptic elements in an interactive simulated environment in real time [4]. VR simulations provide realistic settings that allow for the acquisition of new skills, reinforcement of existing skills, and application of cognitive concepts. The VR's high degree of interaction and realism has been associated with knowledge retention and translation. VR has been explored for nurse education in various contexts, and in crisis training provides engagement, immersion, and realism to participants [5,6].

Key features of virtual learning include 1) immersive and realistic 3D environments in which the users can actively interact with the simulated elements of the system (i.e., pass surgical instruments to the surgeon); 2) real-time visual and/or auditory feedback; and 3) a safe learning environment [3,6].

The purpose of this study was primarily to develop a VR simulation that could replicate a pediatric neurosurgery craniotomy procedure reflecting a real OR scenario, including surgical equipment, an interdisciplinary component (surgeons, nurses, and anesthesiologists), and the surgical procedural sequence of a craniotomy; secondary purpose was to assess OR nurses’ confidence in assisting craniotomy procedures as scrub nurses before and after the VR simulation. We also noted any issues with the hardware or software.

## Materials and methods

Ethics

The study received approval as a quality initiative project at the Hospital for Sick Children, Toronto. Written informed consent was obtained from participants. The project was started in August 2018 and completed in April 2019.

VR model development

A pediatric craniotomy procedure was replicated using VR technology. The development of the VR craniotomy involved a collaborative partnership between education, content, and technology experts within the Hospital for Sick Children, Toronto. Content experts included the Clinical Orientation Support nurse (COSN), who is also specialized in neurosurgery. The content expert identified the domain of learning and ensured that the simulation was applicable and realistic. The education expert included the Inter-professional Education Specialist (IES) for the OR to support knowledge construction and promote reflection and assessment of learning objectives. Technology experts include the Department of Collaborative Human Interactive Immersive Laboratory to help develop an immersive, interactive, and realistic scenario. All departmental experts engaged in continuous consultation throughout the entire development process to ensure that the learning objectives of the VR simulation were relevant, achievable, and measurable. Elements that were considered in the development of the virtual scenario included clinical activities, challenges, redirection, narration, and evaluation. The integration of all these elements has been shown to effectively facilitate learning and retention of content [1]. 

Study participants

Study participants were OR nurses with varying degrees of perioperative nursing experience and knowledge working at The Hospital for Sick Children, Toronto. Participation in the study was voluntary. 

Simulation application and nurses’ confidence evaluation

**Figure 1:**
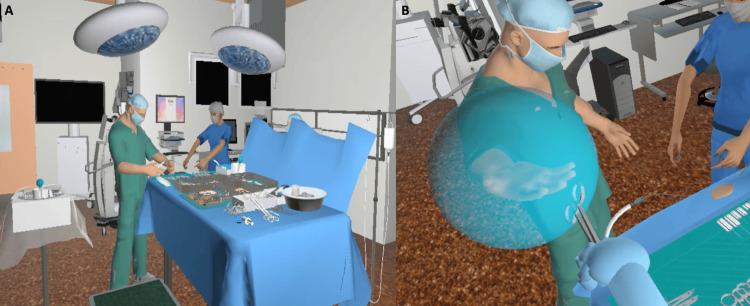
NeuroVR app (A) VR simulation scenario showing a surgical table for craniotomy procedures and surgeons. (B) The frame of the VR simulation session, where a scrub nurse is passing surgical instruments to the surgeon and depicting the target area (Image credit: Clyde Matava). VR, virtual reality

The virtual simulation re-created a craniotomy procedure on a pediatric patient where the user was embedded in the virtual scenario as the scrub nurse (Figure 1). The VR simulation used the VR method of a head-mount display system (Oculus Rift VR, Meta Inc, Menlo Park, California, USA) that generates an immersive environment on a small screen that is close to the user’s eyes. Within the scenario, a surgeon would be performing a craniotomy on a pediatric patient with the user assisting as the scrub nurse. The simulated procedure was 25 minutes long. This included the key steps that involve the actions and expertise of a nurse scrubbed into the case. The real procedure can last anything from 90 minutes to 6 hours. However, the key steps involving the nurse are repetitious in the real procedure and were included in the VR simulation. 

The users were required to apply their knowledge of a craniotomy procedure to set up the table, handle and pass the appropriate surgical instruments at the surgeon’s request, and anticipate the needs of the surgeon and patient throughout the procedure. Feedback was provided during each step with opportunities for redirection, as well as consideration for timely completion of the procedure. Additionally, an overall evaluation of performance was provided upon completion of the scenario that included a breakdown of critical points. 

Self-confidence among OR nurses to assist in craniotomy procedures was explored pre- and post-VR training sessions with a questionnaire ideated by the authors evaluating knowledge relevant to assisting craniotomy procedures with seven items (knowledge of surgical sequences of a craniotomy, knowledge of the instruments required at each stage of a craniotomy, recognize the signs of an intra-operative bleeding, anticipate the needs of the surgical team during an intra-operative cranial bleeding, identify the hemostatic agents required during a bleed, and assist in a craniotomy as a scrub nurse) rated with a five-item scale (have not had any experience, not at all comfortable with the procedure, somewhat comfortable with the procedure, moderately comfortable with the procedure, and extremely comfortable with the procedure).

Each participant’s session was carried out in an old OR used for simulation training. All equipment in this room was cleared to create a safe "six foot by six foot" space for using the VR equipment. Participants then wore the VR headset and were oriented into the virtual OR by a virtual assistant built into the neuroVR application. The virtual orientation lasted three minutes during which participants learned how to move and pick up surgical instruments in the virtual room. Once they had achieved all the orientation goals, they were advanced to the actual virtual neurosurgery scenario. A research assistant remained present throughout to ensure their safety and to troubleshoot any issues with the software or hardware.

Statistical analysis

Descriptive statistics were used to summarize the data questionnaire results. The introduction to the OR environment includes rotations through different services including neurosurgery. As this is a specialized area it may require two to three years for a nurse to gain the required exposure and expertise in neurosurgery. As such we divided the nurses into those with under two years versus those with three years’ experience in the OR. Categorical variables are presented as frequencies and percentages. This was a feasibility and acceptance study with no planned tests for significance. 

## Results

In total, seven OR nurses participated in the study. Three nurses had between zero and two years of experience; three nurses had between three and five years of experience; one nurse had more than 10 years of experience. The post-VR sessions questionnaires showed an increase in positive answers “extremely comfortable with the procedure” and “moderately comfortable with the procedure” compared to pre-VR sessions (Table 1).

**Table 1:** Self-reported confidence of nurses in assisting craniotomy procedures before and after VR simulation session Positive answers: sum of “extremely comfortable with the procedure” and “moderately comfortable with the procedure” answers.

	Positive answers Pre-VR	Positive answers Post-VR
Knowledge of the instruments required at each stage of a craniotomy	4 (57.2%)	5 (71.5%)
Recognize the signs of an intra-operative bleed	3 (42.9%)	5 (71.5%)
Anticipate the needs of the surgical team during an intra-operative craniotomy bleed	4 (57.2%)	6 (85%)
Knowledge of a craniotomy set up	4 (57.2%)	4 (57.2%)
Identify the hemostatic agents required during a bleed	6 (85.7%)	6 (85.7%)
Assist in a craniotomy as the scrub nurse	4 (57.2%)	6 (85.7%)

**Figure 2:**
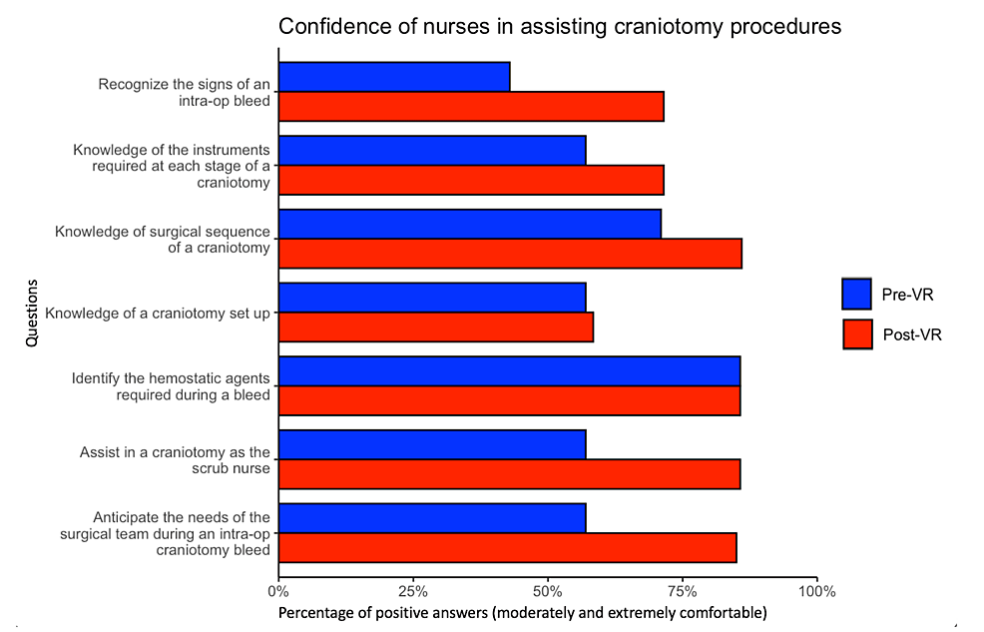
Self-reported confidence in nurses assisting in craniotomy procedures Barplot showing the percentage of positive answers (“extremely comfortable with the procedure” and “moderately comfortable with the procedure”) before and after VR sessions.

In all items except for “identify the hemostatic agents required during a bleed” no difference was noted. The items with the highest increase in positive answers were “recognize the signs of an intra-op bleed” (28.6%) and “assist in a craniotomy as the scrub nurse” (28.5%). Figure 2 shows the differences in positive answers pre- and post-VR sessions. There were zero incidents of software or hardware malfunction. 

## Discussion

The results of this study show the acceptance and positive effect of VR simulation on the confidence of the perioperative nurses at our institution in knowledge for assisting with craniotomy procedures as a scrub nurse.

In particular, the items “recognize the signs of an intra-op bleed” and “assist in a craniotomy as the scrub nurse” showed the greatest increase in confidence; this can be linked to the fact that both the skills and experience, not just the theory of such events, are required for the confidence of the skill being adequately acquired. Conversely, the identification of hemostatic agents required during a bleed has not improved with the VR session, likely because this is a part of theoretical knowledge and is not neurosurgery-specific knowledge, since bleeding may happen in all surgeries while on the other hand, hemostatic agents are not commonly used in neurosurgery. 

Our results are aligned with other studies exploring the role of VR in nurse education. In particular, a study by Permana et al. on VR simulation for nursing care in patients with acute respiratory infections assessed pre- and post-knowledge on the topic with a test on the control (traditional training) and intervention group (VR simulation); results have demonstrated a positive impact on knowledge on the VR simulation group [7]. Another study on a VR scenario designed to train scrub nurses for the preparation of the instrumentation table for a craniotomy in the OR showed high acceptability among participants with no differences between experienced and non-experienced scrub nurses, showing that VR training could be useful both for initial training and for maintaining expertise [8]. 

The training model for nurses with VR thus could create equal and safe learning opportunities for all OR nurses and offer solutions to logical challenges associated with perioperative education and have good acceptability. 

Limitations

The main limitation of this study is that the small sample size and study design were for feasibility and acceptance and did not allow testing for statistical significance. A prospective study with a larger sample size and further studies to test knowledge acquisition with VR versus a traditional learning model should be planned to confirm the positive signal of this study. A limitation inherent to this specific VR simulation model is the absence of haptic qualities. This limitation has been overcome by some VR models that included sophisticated haptic devices in the simulation, such as a wrist-force-feedback-module that simulated the catheter insertion drag in an endotracheal suction simulation or tactile sensory gloves in a urinary catheterization simulation [9-11]. However in our simulated context (scrub nurses), haptic qualities are less important than in clinical procedures learning as the skills are related to picking up, passing, and placing. Furthermore, it is likely that the knowledge acquisition during VR simulation can be safely and effectively transferred into real clinical practice.

## Conclusions

VR simulation session is an acceptable model to train OR nurses for the scrub nurse role in craniotomy procedures. VR simulation is a practical learning strategy for clinical situations that may occur inconsistently in real-time practice. The application of virtual reality can be used to train both new and experienced nurses to proficiently function as part of the perioperative team.

## References

[1] Farra SL, Miller ET, Hodgson E (2015). Virtual reality disaster training: translation to practice. Nurse Educ Pract.

[2] Wilson G (2012). Redesigning OR orientation. AORN J.

[3] Eriksson J, Lindgren BM, Lindahl E (2020). Newly trained operating room nurses' experiences of nursing care in the operating room. Scand J Caring Sci.

[4] Schmidt B, Stewart S (2009). Implementing the virtual reality learning environment: second Life. Nurse Educ.

[5] García-Pazo P, Pol-Castañeda S, Moreno-Mulet C, Pomar-Forteza A, Carrero-Planells A (2023). Virtual reality and critical care education in nursing: a cross-sectional study. Nurse Educ Today.

[6] Sharon L, Farra ETM (2013). Integrative review: virtual disaster training. J Nurs Educ Pract.

[7] Permana RH, Suryani M, Paulus E, Rakhmawati W (2020). The impact of virtual reality simulation on cognitive achievement of nursing students. Indonesian Nurs J Educ Nurs Clinic.

[8] Bracq MS, Michinov E, Arnaldi B, Caillaud B, Gibaud B, Gouranton V, Jannin P (2019). Learning procedural skills with a virtual reality simulator: an acceptability study. Nurse Educ Today.

[9] Komizunai S, Colley N, Konno A (2020). An immersive nursing education system that provides experience of exemplary procedures from first person viewpoint with haptic feedback on wrist. 2020 IEEE/SICE International Symposium on System Integration.

[10] Butt AL, Kardong-Edgren S, Ellertson A (2018). Using Game-Based Virtual Reality with Haptics for Skill Acquisition. Clin Simul Nurs.

[11] Alam F, Matava C (2022). A new virtual world? The future of immersive environments in anesthesiology. Anesth Analg.

